# Ablation of unmappable ventricular parasystole originating from the right ventricular outflow tract: a case report

**DOI:** 10.1186/s12872-018-0992-0

**Published:** 2019-01-05

**Authors:** Huan Wang, Lihong Wang

**Affiliations:** 1 Department of Cardiology, Zhejiang Provincial People’s Hospital, Hangzhou, 310014 China; 2 Department of Cardiology, People’s Hospital of Hangzhou Medical College, Hangzhou, 310014 China

**Keywords:** Ventricular arrhythmia, Catheter ablation, Right ventricular outflow tract

## Abstract

**Background:**

When the coupling interval is matched, ventricular parasystole can form a stable fusion QRS complex with sinus rhythm. Ablation of a fusion QRS complex has been rarely reported and is unexpectedly difficult.

**Case presentation:**

We describe a case of ventricular parasystole from muscle sleeves of the right ventricular outflow tract. The patient was a 54-year-old woman who was admitted to the hospital because of frequent palpitations for 3 months. Anti-arrhythmic drugs had been ineffective, and she had no history of cardiovascular disease. Because the fusion QRS complex interfered with the conventional mapping technique, we could not eliminate the ventricular parasystole successfully.

**Results and conclusions:**

Finally, we used the reversed U curve method and found that the source of ventricular arrhythmia was in the right cusp according to the special local potential. A fusion QRS complex formed by ventricular parasystole and nodal ventricular activation make mapping and ablation difficult. The special local potential was the only evidence available to confirm the target of ablation satisfactorily.

## Background

Premature ventricular contraction (PVC) is among the most common types of arrhythmia encountered in clinical practice, while the right ventricular outflow tract (RVOT) is the most common site for idiopathic PVC origin [1, 2]. PVCs from the RVOT are considered suitable for catheter ablation with low complication rates and high success rates [3].

Ventricular parasystole (VP) is defined as a dual rhythm wherein an ectopic pacemaker is in some way protected from the impulse of the sinus pacemaker. It is not rare in the clinical setting, especially in disease heart, and it is easily confused with simple PVC [4]. When the coupling interval is matched, VP can form a stable fusion QRS complex with sinus rhythm. We herein describe such a case of VP from the RVOT in a healthy person. As far as we know, ablation of a fusion QRS complex composed of VP and sinus rhythm has not been reported before.

## Case presentation

**Fig. 1:**
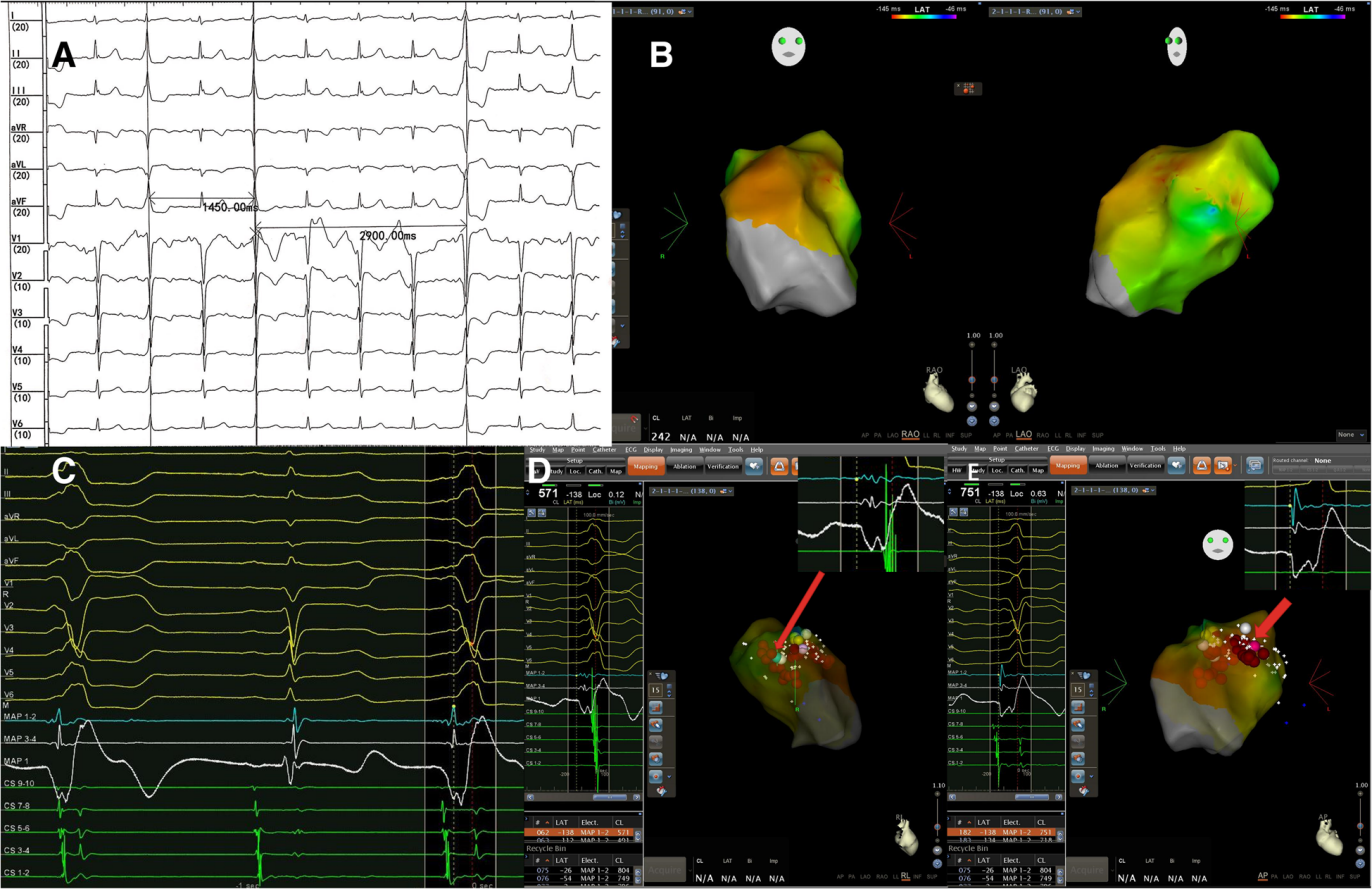
**a** Twelve-lead surface electrocardiogram. Premature ventricular contractions (PVCs) demonstrate varying coupling intervals, and the morphology of PVCs includes a complete left bundle branch block, inferior frontal axis, and precordial lead transition zone >V3. The QRS in lead I is positive, and the R-wave in lead II is more than that in lead III. The coupling intervals of ectopic beats vary with multiple coupling intervals. **b** Activation mapping of ventricular parasystole (VP) shows no distinctively early focus. The anterior free wall of the right ventricular outflow tract (RVOT) is slightly precedent. **c** Bipolar and unipolar electrocardiograms during two adjacent VPs. **d** The fusion degree of VP and the nodal rhythm is different between the two VPs, and the morphologies of the QRS complexes are different. **b** Three-dimensional image and local bipolar and unipolar electrocardiograms of the target located at the middle free wall of the RVOT (green point); ventricular activation on the unipolar electrocardiogram demonstrates a QS morphology. **e** Three-dimensional image and local bipolar and unipolar electrocardiograms of the target located at the anterior free wall of the RVOT (red point); ventricular activation on the unipolar electrocardiogram also demonstrates a QS morphology

The patient was a 54-year-old woman who was admitted to the hospital because of frequent palpitations for 3 months. Anti-arrhythmic drugs including mexiletine, propafenone, and metoprolol had been ineffective. She had no history of cardiovascular disease. PVCs detected by surface 12-lead electrocardiography (ECG) had the following morphology: a complete left bundle branch block, inferior frontal axis, and precordial lead transition zone >V3. The QRS in lead I was positive, and the R-wave in lead II was higher than that in lead III. The findings suggested that the PVCs were from the free wall of the RVOT. Most of the time, the ectopic beats demonstrated bigeminy with stable coupling intervals, but sometimes, the coupling intervals varied and multiplied. These findings implied that the PVCs were actually VP (Fig. 1a). Twenty-four-hour dynamic ECG showed more than 32,000 PVCs.

After withdrawal of anti-arrhythmic drugs for 5 or more half-lives, the patient underwent an electrophysiological evaluation. Both bipolar and unipolar electrograms were recorded by a LEAD-7000 EP Recording System (Sichuan Jinjiang Electronic Science and Technology Co., Chengdu, China) filtered at 30–500 Hz and 0.05–500 Hz, respectively. Three-dimensional electromagnetic mapping (CARTO, Biosense Webster, Diamond Bar, CA) and ablation were performed via a 7-French saline-irrigated ablation catheter with a 3.5-mm distal electrode and 2–5-2 mm interelectrode spacing. Activation mapping and pace-mapping were combined to identify the origin of VP. Activation times were assigned based on the earliest rapid downstroke of the unipolar signal (fastest dV/dt) correlating with the first sharp peak of the bipolar electrogram. The reference line was a sharp peak of QRS in lead II.

As a result, activation mapping in the RVOT region was performed and failed to indicate the earlier activation region (Fig. 1b). Pace-mapping also failed. The key reason for failed mapping was fusion of VP and nodal QRS. In this case, all ventricular activations during VP had two components. The degree of fusion was unstable, which accordingly made the morphology of QRS variable, as detected by surface ECG. Therefore, there was no stable reference for activation mapping (Fig. 1c). The precedence of local activation was unmeasurable. For the same reason, pace-mapping was also unavailable because there was no QRS template of pure VP activation. We tried to find the source of VP according to the morphology of the V-wave by unipolar ECG. Two target points were confirmed in the anterior and middle regions of the RVOT free wall, respectively (Fig. 1d, e). However, ablations were not effective at these positions.

## Results and conclusions

**Fig. 2:**
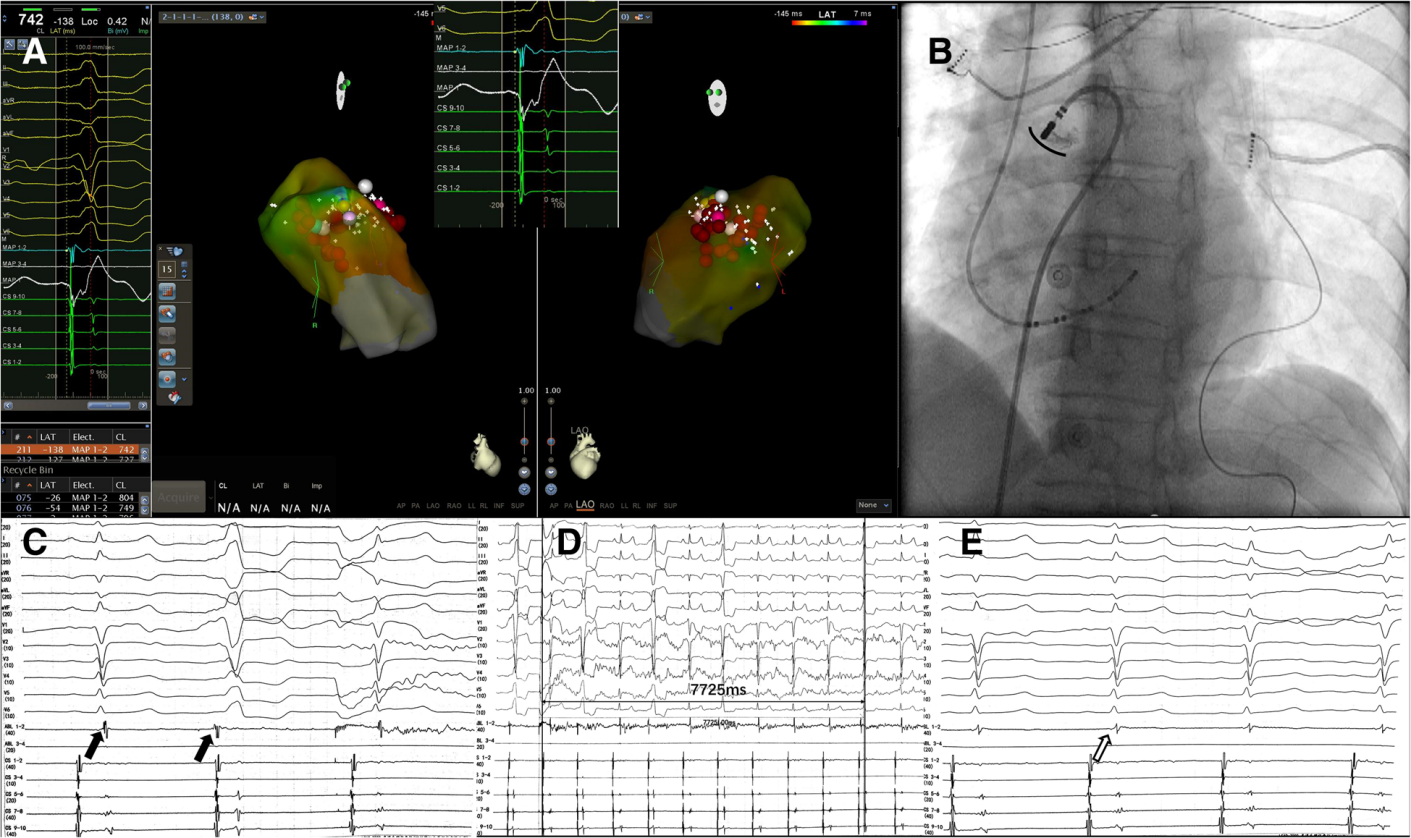
**a** The final target in the right cusp (RC) (white point). Ventricular activation on the unipolar electrocardiogram has a sharp QS morphology. **b** Angiogram of the final target via the reversed U curve method. The black curve shows the bottom of the RC. **c** Local bipolar potential at the final target. There is a blunt far-field activation followed by a sharp near-field potential during sinus rhythm (the first black arrow). The relationship between the two signals is reversed during ventricular parasystoles (VPs) (the second black arrow). **d** After 7 s of ablation (30 W, 43 °C), VPs are terminated. **e** Local bipolar potential at the final target. After catheter ablation, the sharp potential is eliminated (the blank arrow)

A reversed U curve of the ablation catheter was used to map the region above the pulmonary sinus cusp (PSC). Target 3 was found in the bottom of the right cusp (RC) (Fig. 2a, b). The V-wave detected by local unipolar ECG was QS-shaped, and there was a fragmented high frequency potential after the bipolar V-wave in sinus rhythm, and potential reversal occurred during PVC (Fig. 2c). After 7 s of ablation, the premature beat was terminated (Fig. 2d, e).

When physicians say ‘unmappable arrhythmia’, they are usually referring to cases in which conditions interfere with the mapping procedure, such as unsustained arrhythmia or arrhythmias from multiple sources or with multiple exits. Herein, we described a different reason that caused the arrhythmia to be unmappable: a case of VP from the RVOT, which was proven to be muscle sleeve-related and formed a fusion wave with nodal QRS. Ablation of a ventricular arrhythmia like this has not been reported before.

VP was recognised based on the following three electrocardiographic signs: (1).

marked variation in the coupling of the ectopic beats; (2) regular appearance of the ectopic beats; and (3) appearance of combination, fusion, or summation beats. [4] Accordingly, a diagnosis of VP was clear in this case. Although VP is per se rarely hemodynamically relevant, it could induce symptomatic ventricular tachycardia and fibrillation in some situations; thus, radiofrequency ablation is amenable in the case of failure of anti-arrhythmic agents [5, 6].

Because fusion of VP and nodal rhythm was unstable in this case, ablation of such an changeable fusion QRS complex was difficult. First, in ablation of arrhythmia from an ectopic focus, activation mapping is necessary and invaluable. In essence, activation mapping compares the local ventricular stimulation with a stable reference; thus, the operator can obtain relative precedence of activation at a different focus [1, 7]. In this case, as the degree of fusion varied, it caused activation mapping to lose a stable reference; therefore, the precedence of local activation was unmeasurable [8], and the activation map could not precisely indicate the source of VP. It looked like the PVCs had multiple exits, but it was completely different in nature. In PVCs with multiple exits, there can be several but finite morphologies of the QRS complex. The QRS morphology was consistent with the location of the exit. In most cases, there is always a dominant morphology of the QRS complex that can be mapped and ablated. In contrast, the QRS morphology in our case was confusing, and no dominant morphology of the QRS complex could be used for mapping and ablation. Nakahara and colleagues [9] reported a case of ablation of VP. They concluded that they could ablate both VP and VPC with the fixed coupling interval using the earliest potential in the ventricle. However, there was no fusion component in VP in their paper.

Second, pace-mapping is an accessory tool that is often used after activation mapping has identified the point of interest. It is also useful in cases of rare or absent PVCs. The premise of pace-mapping is that in a focal arrhythmia, pacing at the site of ectopy produces a surface QRS in 12 leads that is nearly identical to clinical arrhythmia [1, 7]. However, in this case, because the target VP was fused with nodal QRS, the pure activation template of the ectopic focus was absent. Hence, the value of pace-mapping was very limited here.

As concluded above, fusion interfered with the mapping techniques, making mapping challenging. The fundamental solution to this problem is to separate VPs from nodal QRS, but how? As a previous paper reported, the coupling interval of VP is affected by various factors, such as sinus rhythm [10], position [11], and drugs [12]. In this case, we tried various methods, such as an intravenous injection of isoproterenol and atrial pacing. Those methods could indeed change the natural frequency of VP; however, they could only influence the degree of fusion, but not completely separate VP and the nodal rhythm. Considering the effect on VP, intravenous injections of esmolol or verapamil were excluded as options, although those drugs might reduce nodal ventricular activation.

Understanding of the mechanism and clinical characteristics of PVCs from the RVOT has improved in recent years. As reported by previous papers, the muscle sleeve above the pulmonary artery is clearly associated with PVCs from the RVOT, which could be an ideal target for ablation. Liao [13] and Zhang [14] summarised the characteristics of PVCs above the PSC. In this case, the location of VP was proven to be located at the bottom of the RC by angiography. Most importantly, blunt far-field activation followed by a sharp near-field potential was observed at the target sites during sinus rhythm. The relationship between the two signals was reversed during VPs. The special potential was associated with muscle sleeve electrophysical activation [13, 14]. It was proven that the VP originated from the muscle sleeve of the pulmonary artery. As concluded by Zhang, recording of the earliest sharp potential can predict successful ablation in 100% of patients [14]. When the conventional mapping method was invalid, the final target was determined by the special local potential.

Finally, we had a few thoughts on this case. First, because of catheter stability and contact force, ventricular arrhythmias from the free wall of the RVOT would be challenging to treat with the conventional ablation method. In those cases, the reversed U curve method in PSC is a better choice [15]. In the present case, if we started with the reversed U curve method, mapping and ablation would have been easier. However, this method could not fundamentally change the difficult situation of mapping. Second, if a fusion QRS complex originates from a site without special local potential, how can physicians find the target? In informal peer communication, it was suggested that physicians can find the earliest target point via a high-density mapping catheter such as the PentaRay (Biosense-Webster), which can be used to identify activation of multiple local points in one heartbeat.

Ablation of a fusion QRS complex formed by VP and nodal rhythm is challenging because fusion interferes with the mapping techniques. In this case of VP from the RVOT, we used the reversed U curve method and found the special earlier sharp potential in the RC, which might be the only clue to finding the target satisfactorily.

## Abbreviations

ECG: Electrocardiography
PSC: Pulmonary sinus cusp
PVC: Premature ventricular contraction
RC: Right cusp
RVOT: Right ventricular outflow tract
VP: Ventricular parasystole
